# Spontaneous tendon rupture in a patient with systemic sclerosis: a case report

**DOI:** 10.1186/s12891-022-05967-6

**Published:** 2022-11-22

**Authors:** Cong Lin, Jun Shen, Zhixing Jiang, Yi Cheng, Yundong Shen, Guoqiang Ren, Wendong Xu, Weiguo Wan, Ling Cao, Hejian Zou, Xiaoxia Zhu

**Affiliations:** 1 Division of Rheumatology, Huashan Hospital, Fudan University, Shanghai, China; 2 Institute of Rheumatology, Immunology and Allergy, Fudan University, Shanghai, China; 3 Department of Hand Surgery, Huashan Hospital, Fudan University, Shanghai, China; 4 Department of Ultrasound in Medicine, Huashan Hospital, Fudan University, Shanghai, China; 5 Department of Pathology, Huashan Hospital North, Fudan University, Shanghai, China

**Keywords:** Systemic sclerosis(SSc), Tendon, Rupture, Case report, Rheumatic disease

## Abstract

**Background:**

Systemic sclerosis (SSc) is an incurable autoimmune disease characterized by progressive skin fibrosis and organ failure. Tenosynovitis is a common musculoskeletal manifestation, but tendon rupture has seldom reported in SSc.

**Case presentation:**

We present a rare case of a 49-year-old female with SSc who has suffered from bilateral tendon rupture of the fourth and fifth digits with positive antinuclear antibody (ANA) and anti-centromere B antibody, but negative rheumatoid factor in serum. In the extensor tendons of the patient’s hands, inflammation, edema, hypertrophy and tendon interruption were detected with ultrasound and magnetic resonance imaging(MRI). Tendon transfer repair surgery was performed and 10 mg/week methotrexate was then used in this patient. Her hand function was improved well with methotrexate and rehabilitation treatment postoperatively.

**Conclusions:**

Early detection of tenosynovitis is necessary to prevent tendon rupture in SSc patients. Ultrasound and Magnetic Resonance Imaging appear to be useful examinations for evaluating tendon pathology for early detection.

## Background

Systemic sclerosis (SSc) is an incurable autoimmune disease characterized by immune abnormalities, inflammation and fibrosis [1]. It involves progressive skin fibrosis and organ failure [2]. Tenosynovitis is a common musculoskeletal manifestation and has been determined to be an important predictor of the progression of SSc [3]. However, tendon rupture has seldom been reported previously. Here, we present a case of a woman who developed complete extensor tendon rupture of the little and ring fingers due to progressive SSc.

## Case presentation

**Fig. 1:**
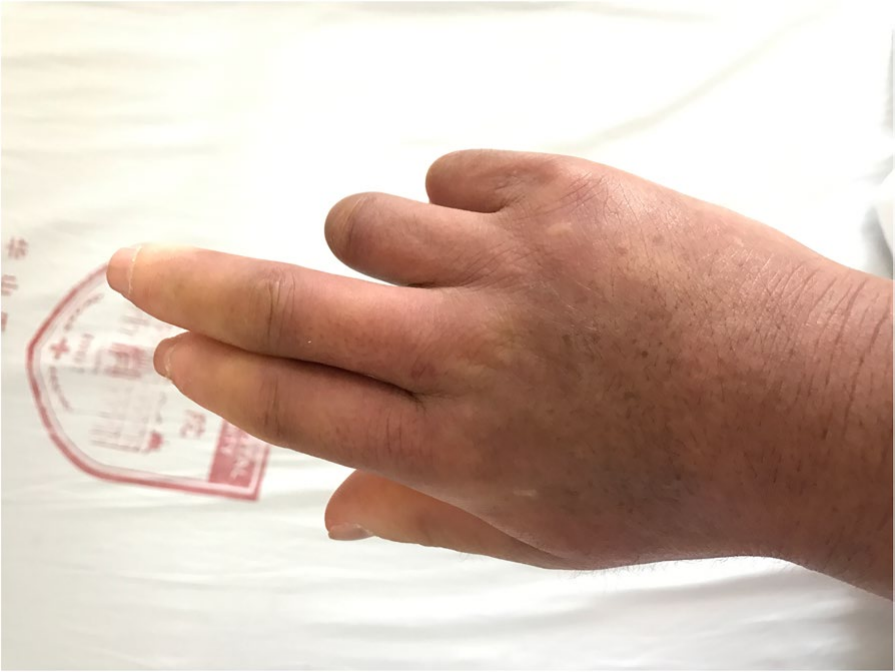
The patient was unable to extend her fourth and fifth fingers of her right hand

The patient was a 49-year-old female farmer who had suffered from Raynaud’s phenomenon and sclerodactyly for 30 years. She experienced tightening of her fingers, face and forearm and then gradually developed restricted movement of both hands. She denied any treatment except for intermittent traditional Chinese treatment to diminish the symptoms. She did not suffer from arthralgia, acid reflux, dysphagia, cough, or shortness of breath. Importantly, the patient noted the abrupt onset of a painless inability to extent the fifth digit of her left hand in June 2015. One month later, the same symptom developed in the fourth digit. She was then diagnosed with tendon rupture and underwent tendon repair in the local hospital. In October 2016, the same symptom happened to the fifth and fourth digits of her right hand (Fig. 1). From then on, she suffered from a disorder of the fourth and fifth digits of both hands. On physical examination, the patient was unable to actively extend the little and ring fingers at the metacarpophalangeal joints of both hands.

**Fig. 2:**
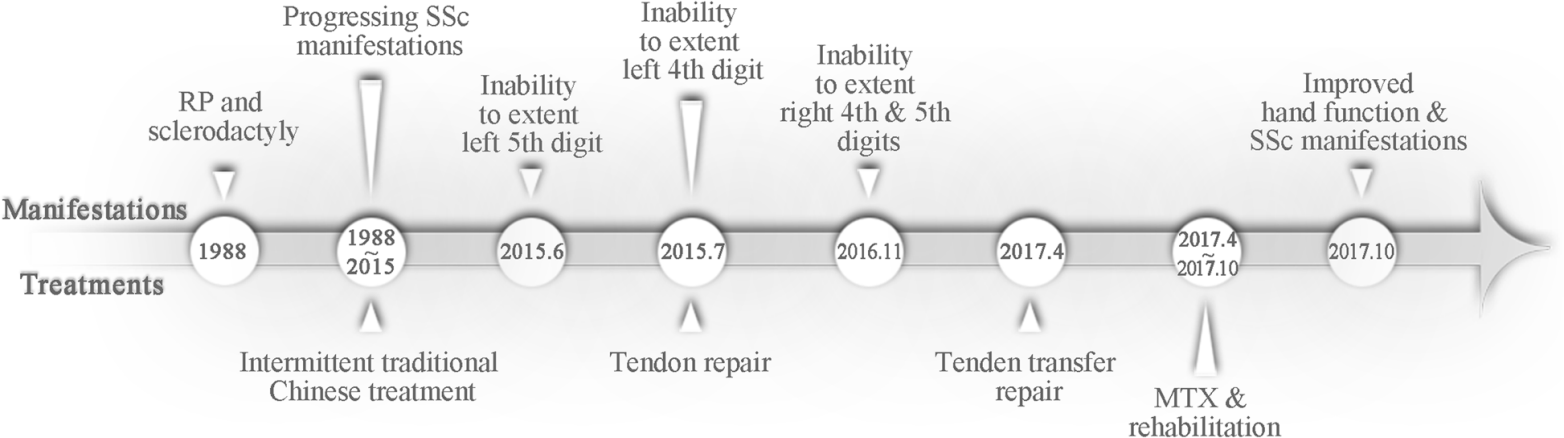
Timeline of symptoms and treatments for this patient. RP = Raynaud’s phenomenon; SSc = systemic sclerosis; MTX = methotrexate

Results of laboratory tests showed an antinuclear antibody (ANA) count of 1:3200 with a centromere pattern and positive anti-centromere B antibody. The erythrocyte sedimentation rate(ESR) was 37 mm/hour, and serum C3 and C4 levels were slightly reduced at 0.70 g/L and 0.23 g/L. Rheumatoid factor, Scl-70, CCP antibody and other related antibodies were negative. No abnormality was shown on chest computed tomography (CT) scan or heart echocardiography. She was diagnosed as diffuse cutaneous SSc(dcSSc) with bilateral extensor tendon rupture of fourth and fifth digits. After successful tendon transfer, the function of the fourth and fifth digits of both hands was gradually improved with methotrexate and rehabilitation treatment. After half a year, ESR was decreased as 22 mm/hour, and she felt improvement of fingers tightening and hands movement restriction. A timeline for the patient is shown in Fig. 2.

Carpal tunnel syndrome was excluded because no abnormality of nerve conduction was detected by electromyography examination. Ultrasonography and Magnetic Resonance Imaging (MRI) examination were further used to help diagnose.

**Fig. 3:**
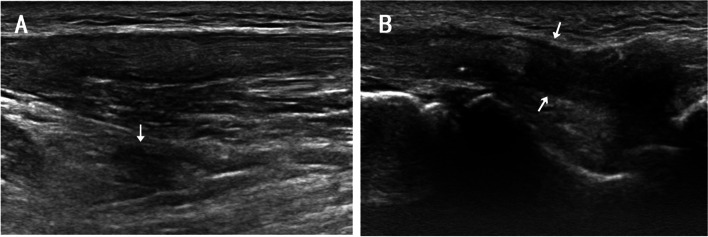
In ultrasonic images, extensor tendon of right ring and little finger was shown ruptured surrounding tissue edema, the proximal end (A) and the distal end (B) were indicated with white arrow

**Fig. 4:**
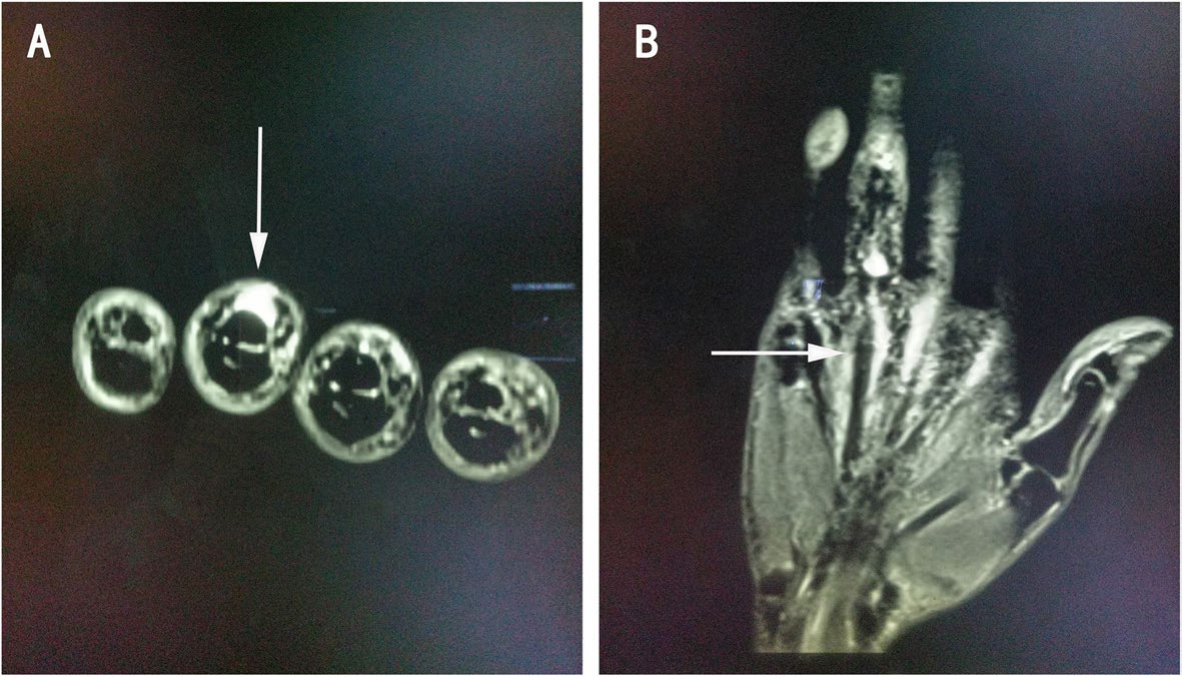
MRI T2-weighted image shows in-continuous high signal in finger extensor tendon (**A**) and edema in the surrounding tissue (**B**)

On the ultrasound image, extensor tendon interruption and surrounding edema was detected in the fourth and fifth fingers of the right hand (Fig. 3). On MRI T2-weighted image, a discontinuous high signal in the tendons of the fourth and fifth fingers and edema in the surrounding tissue were detected (Fig. 4). Both MRI and ultrasound results suggested tendon rupture of the right fourth and fifth fingers with remarkable inflammation in the tendons and surrounding tissue.

**Fig. 5:**
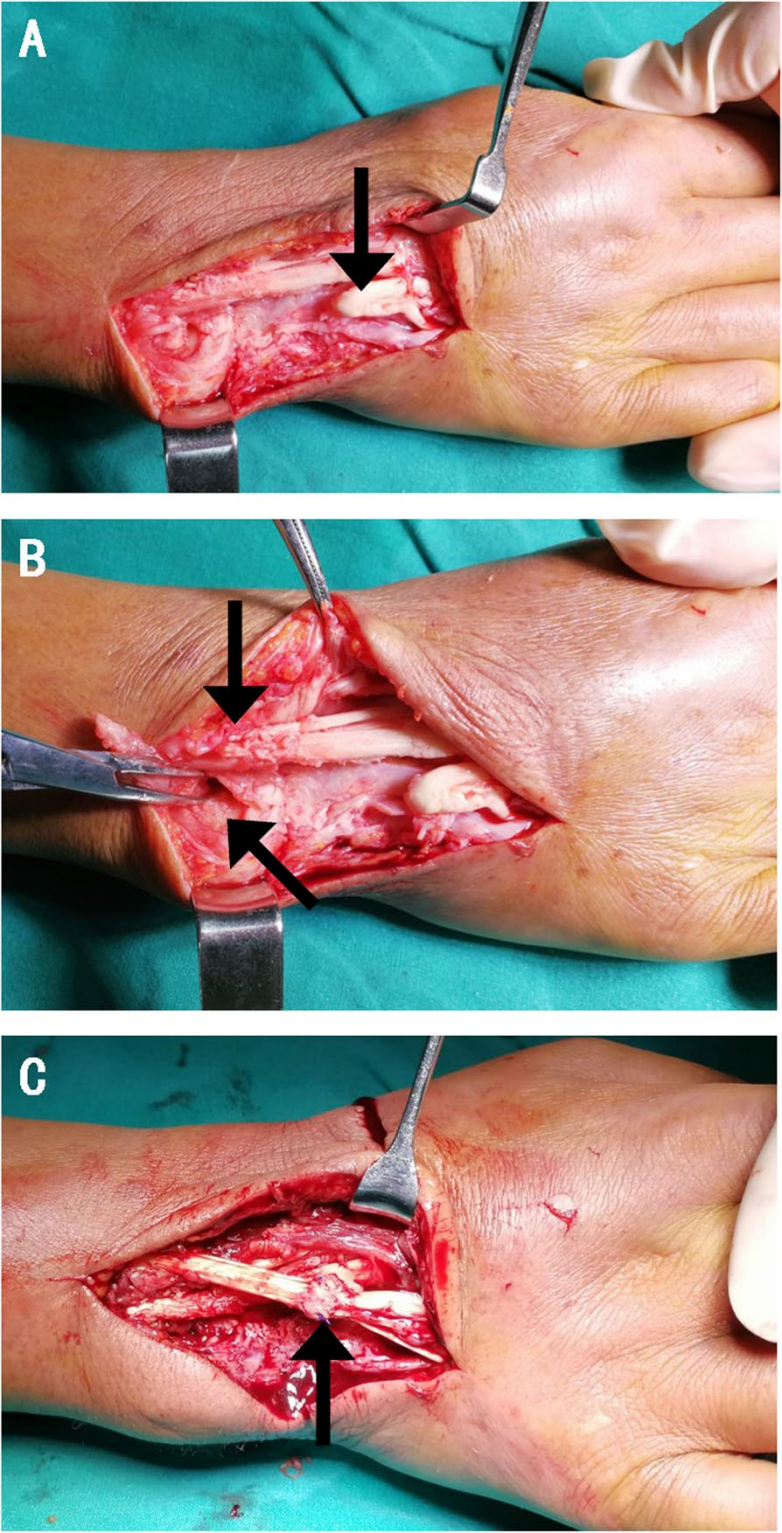
Surgical exploration demonstrating tendon ruptures with proximal and distal ends (**A**), perforation of the dorsal capsule and hairy appearance of the tendon (**B**), performance after tendon reconstruction (**C**)

Based on the symptoms and examinations of this patient, rupture of the fourth and fifth finger extensor tendons was suspected, and a surgical exploration was performed. The ruptured fourth and fifth extensor tendons of the right hand were confirmed during operation. The proximal stumps were located under the extensor compartment retinaculum, and distal stumps lay on the middle of the fourth metacarpal bone. Other tendons showed edematous appearance, indicating chronic inflammatory changes (Fig. 5A). Perforation of the dorsal wrist capsule and instability of the radioulnar joint were shown (Fig. 5B). The tendons lying directly on the distal radioulnar joint had a notably hairy appearance (Fig. 5B). Tendon transfer repair was performed in this patient. The tendon of extensor carpi radialis longus (ERCL) was detached at its insertion at the base of the second finger metacarpal which was transferred and sutured to the distal ruptured tendon stumps via subcutaneous tunnel (Fig. 5C). After surgery, the patient received methotrexate 10 mg/week to inhibit immune inflammation.

**Fig. 6:**
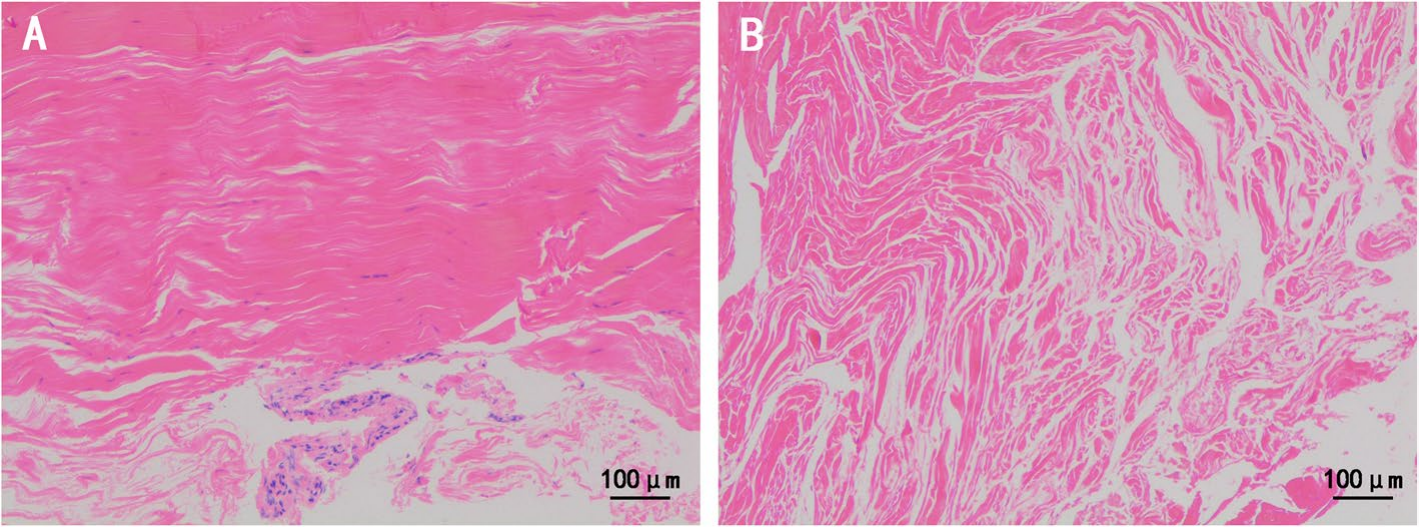
Histopathologic examination of the ruptured tendon in health control (**A**) and the patient (**B**)

Histopathologic examination of the tenosynovium revealed degenerated tendinous tissue with fibrosis of advanced stage and irreversible damage. In a healthy control with tendon rupture by trauma, lines of collagenous fibers and scattering distribution of spindle tendon cells are shown in the pathological section (Fig. 6A). In our SSc patient, the tissue sections demonstrated structural disorder, the proliferation of collagen fibers and the infiltration of fibroblastic tissue. No infiltrated lymphocytes or inflammatory cells were seen in the tissue sections (Fig. 6B).

## Discussion

SSc is a multisystem disease that represents various clinical manifestations besides skin fibrosis. As a previous cross-sectional analysis reported, articular involvement frequently manifests as synovitis, and bursitis is very common in SSc [4]. Patients with SSc often show a loss of flexion at the metacarpophalangeal joints and proximal interphalangeal joints, described as characteristic “claw” deformities of the hands [5]. Our patient’s fifth and fourth digits of the right hand were drooped and completely lost function except for “claw” deformities of her hands. She was finally diagnosed with SSc and tendon rupture. Physicians should be aware that finger drop or wrist drop in SSc may be related to extensor tendon rupture and that prompt evaluation is necessary.

Tendon rupture has been associated with rheumatoid arthritis [6]. Our patient had the typical clinical features of SSc with a positive ANA and anti-centromere B antibody, and the possibility of SSc-RA overlap is excluded due to the absence of arthralgia, negative rheumatoid factor or CCP antibody.

Some SSc patients experience a sense of scraping or rubbing when they move joints, called a tendon friction rub, which is a common manifestation involving the extensor and flexor tendons of the wrist, knee, and ankle [7]. It might be ascribed to fibrin deposition in the tendon’s synovial sheath or the increased thickness of the retinacula [8, 9]. This patient did not display any tendon friction rubs at her metacarpophalangeal areas, wrists, ankles or knees. There was no associated pain or warning symptoms before the tendon rupture. On the ultrasound and MRI images, inhomogenous inflammation and hypertrophy were detected in the extensor tendons of her hands. The tendons presented a remarkable inflammatory appearance in the surgery. It suggested that the inflammation of extensor tendons is one of the primary pathogenic factors in its rupture.

We noticed rupture of both hand extensor tendons of the fourth and fifth fingers in this patient. Similarly, tendon rupture was reported in the fourth and fifth fingers in previous case reports [10, 11]. Here, we supposed that extensor tendon rupture mostly appeared in the fourth and fifth fingers because of the local anatomical structure. Chul-Hyun Cho presented an osteoarthritis case with extensor tendon rupture caused by the dorsal capsular perforation and instability of the ulnar head [12]. Carr AJ and a colleague analyzed 12 osteoarthritis patients with extensor tendon rupture of the ring and little fingers and discovered that all 12 cases showed dorsal capsule perforation of the distal radioulnar joints [13]. In this SSc case, we similarly observed dorsal capsular perforation and distal radioulnar joint instability during the surgery. Contact between the ulnar head and extensor tendons was allowed, and the tendons lying directly over the radioulnar joint appeared to have a notably rough surface. That was strong evidence of mechanical friction of the inflamed tendons with the unstable distal radioulnar joint, which is a potential mechanism of the tendon rupture.

Early detection and treatment are the most important considerations in preventing disease progression and tendon rupture in SSc. It appears that there are few predictive or warning symptoms that precede tendon rupture. Early recognition of tendonitis, arthritis and peri-arthritis depends on ultrasound and MRI. Physical examination could be performed first to assess the tendon function of hands when diagnosis of SSc is confirmed. Then ultrasound and MRI should be applied for patients with pain or tenderness of tendon, or restricted movement of hands to evaluate tendon damage. These imaging examinations will likely provide much more details to predict tendon involvement and specifically the risk of tendon rupture in SSc. Reports have shown that MRI can be used to quantify tendinopathy at the wrist in RA patients [14], and high MRI tendinopathy scores in early disease are predictive of tendon rupture [15]. Compared to MRI, ultrasound performs slightly better at detecting tendon damage including edema, partial tear and complete rupture [16]. An ultrasound scoring system could be applied in SSc for evaluating and following up tendon damage [6].

## Conclusions

When the SSc patient presents with an inability to extend the fingers, especially the little and ring fingers, physicians should consider the possibility of extensor tendon rupture as a complication. Ultrasound and MRI appear to be useful examinations for evaluating tendon pathology for early detection. Further prospective studies are required to explore the clinical value of the two imaging examinations.

## Data Availability

All data generated or analyzed during this study are included in this published article.

## Abbreviations

SSc: Systemic sclerosis
CT: Computed tomography
MRI: Magnetic resonance imaging
ANA: Antinuclear antibody
ESR: Erythrocyte sedimentation rate
ERCL: Extensor carpi radialis longus
